# Usefulness of ^18^F-fluorodeoxyglucose positron emission tomography/computed tomography angiography in a patient with blood culture-negative prosthetic valve endocarditis complicated with perivalvular abscess: a case report

**DOI:** 10.1093/ehjcr/ytz171

**Published:** 2019-10-11

**Authors:** Shiro Miura, Masanao Naya, Takehiro Yamashita, Youhei Ohkawa

**Affiliations:** 1 Department of Cardiology, Hokkaido Ohno Memorial Hospital, 2-1-16-1 Miyanosawa, Nishi-ku, Sapporo 063-0052, Japan; 2 Department of Cardiovascular Medicine, Hokkaido, University Graduate School of Medicine, Kita-15, Nishi-7, Kita-ku, Sapporo 060-8638, Japan; 3 Department of Thoracic and Cardiovascular Surgery, Hokkaido Ohno Memorial Hospital, 2-1-16-1 Miyanosawa, Nishi-ku, Sapporo 063-0052, Japan

**Keywords:** Case report, Endocarditis, Perivalvular abscess, Positron emission tomography

## Abstract

**Background:**

Prosthetic valve endocarditis (PVE) is a life-threatening systemic infection involving a high mortality rate and severe complications, including perivalvular abscess. Early diagnosis and detection of PVE continue to be challenging in clinical settings.

**Case summary:**

A 64-year-old man with a history of mechanical aortic valve implantation 12 years prior was referred to our hospital with a major complaint of high fever and was admitted. Although results of three blood culture tests at admission were negative, transthoracic echocardiography, and transoesophageal echocardiography (TOE) were performed to exclude the possibility of PVE; both, however, were inconclusive. Subsequently, ^18^F-fluorodeoxyglucose positron emission tomography/computed tomography (^18^F-FDG PET/CT) was performed; revealing intense hyper-metabolism above the aortic valve prosthesis with a greater intensity at the posterior end, confirming a diagnosis of aortic PVE complicated with perivalvular abscess.

**Discussion:**

Considering the intermediate suspicion of PVE despite negative TOE and negative blood culture tests, ^18^F-FDG PET/CT can play a central role in diagnosing PVE. However, this new imaging modality often fails to differentiate thrombi, soft atherosclerotic plaques, or foreign body reactions on the surface of prosthetic valves. In this report, we have successfully enhanced the diagnostic accuracy of ^18^F-FDG PET/CT by focusing on perivalvular involvement, which could be a key finding, because intense ^18^F-FDG uptake surrounding the aortic annulus was consistent with the thickened area within the aortic annular region observed in the TOE examinations.

**Learning points**

- Prosthetic valve endocarditis (PVE) can often be a major diagnostic challenge owing to the difficulties in the interpretation of conventional echocardiography, particularly in patients with low-to-intermediate probability for PVE based on the modified Duke criteria.
- We describe a case of PVE complicated by perivalvular abscess where the combination of ^18^F-fluorodeoxyglucose positron emission tomography and computed tomography angiography aided in the diagnosis of PVE and its complications, shedding light on the additional roles of the new imaging modalities.

## Introduction

Definitive diagnosis of prosthetic valve endocarditis (PVE) is clinically challenging because of its varying clinical presentation, microorganisms involved and patient profile. Prosthetic valve endocarditis entails high initial mortality and morbidity rates with a high risk of complications, including perivalvular abscess and fistulae during follow-up and often requires surgical intervention.^1^ Therefore, the diagnosis and management of PVE necessitates a collaborative approach involving cardiologists, infectious disease physicians, cardiac imaging specialists, cardiovascular surgeons, and microbiologists.^2^

Recently, ^18^F-fluorodeoxyglucose positron emission tomography/computed tomography (^18^F-FDG PET/CT) has been advocated as an alternative diagnostic imaging modality for PVE, particularly when echocardiography and blood culture results are inconclusive in reaching a definitive PVE diagnosis.^3^ Furthermore, ^18^F-FDG PET/CT can detect distant emboli, metastatic infections and perivalvular involvement along with coronary anatomy when combined with CT coronary angiography.^4^ Additionally, complementary information obtained from less invasive tests are vital for patients with PVE who require early surgery. Herein, we present a challenging case of PVE complicated with perivalvular abscess.

## Timeline

**Table:** 

12 years before admission	25-mm mechanical aortic valve implantation owing to severe aortic regurgitation.
3 days before admission	Fever at night and general fatigue initiated.
Day of admission	Initial transthoracic echocardiography revealed no dysfunction of the mechanical aortic prosthesis, and no embolic events were detected on the whole-body computed tomography (CT). Three blood culture tests were performed and intravenous antibiotic therapy with ceftriaxone (2 g/day) was initiated.
Hospital Day 5	Transoesophageal echocardiography was inconclusive and fever persisted.
Hospital Day 8	All blood culture tests were negative. ^18^F-fluorodeoxyglucose (FDG) positron emission tomography/CT revealed considerable uptake of ^18^F-FDG surrounding the aortic annulus; intensive antibiotic therapy was initiated with intravenous ampicillin (8 g/day) and gentamicin (180 mg/day).
Hospital Day 14	The infected prosthetic valve was surgically replaced with a 25-mm bioprosthesis with extensive debridement of perivalvular abscess.
Hospital Day 51	The patient was discharged well.
6 months after discharge	A 6-month of outpatient oral antibiotic therapy with amoxicillin (750 mg/day) was completed without any recurrences.

## Case presentation

**Figure 1:**
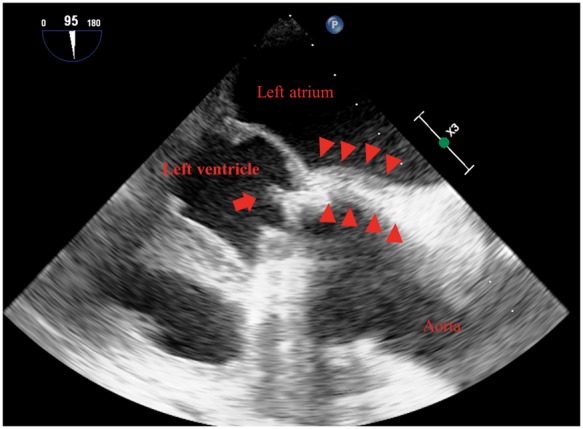
Transoesophageal echocardiography showing a thickened area along the aortic root behind the non-coronary sinus of Valsalva (arrowheads) and a mobile echogenic mass attached to the prosthetic valve leaflet (arrow).

**Figure 2:**
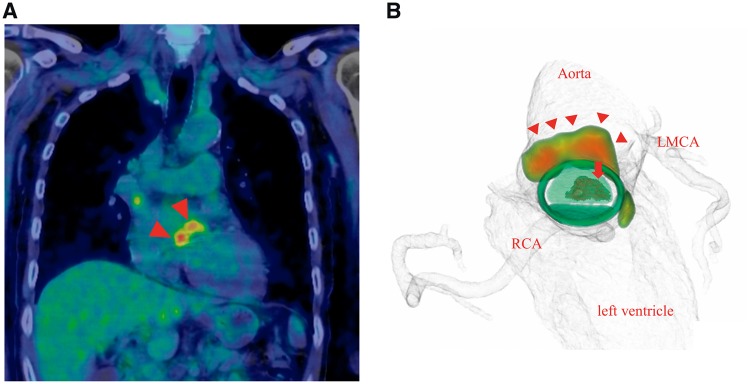
(*A*) ^18^F-fluorodeoxyglucose positron emission tomography/computed tomography showing significant ^18^F-fluorodeoxyglucose uptake (arrowheads) at the level of the mechanical aortic valve with inhibition of the rest of the cardiac metabolism, indicating perivalvular abscess. (*B*) The combination of ^18^F-fluorodeoxyglucose positron emission tomography and computed tomography angiography provides additional information regarding the accurate location of the vegetation behind the valve surface (arrow) and distribution of the affected perivalvular tissue (arrowheads), in addition to characterizing the coronary arteries, aortic valve, root, and ascending aorta. LMCA, left main coronary artery; RCA, right coronary artery.

**Figure 3:**
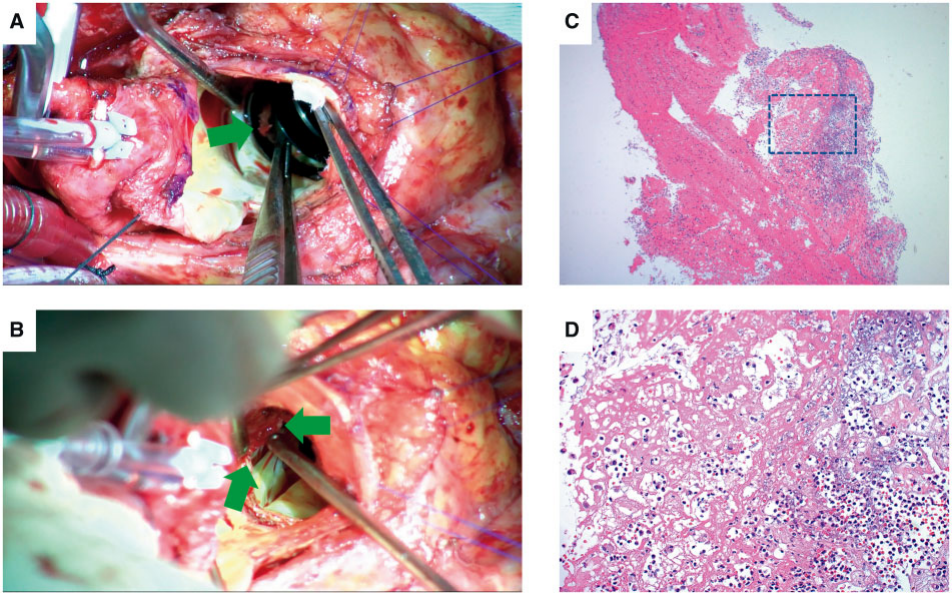
(*A*) Visualization of the mobile vegetation attached to the aortic mechanical prosthesis (arrow). (*B*) Surgical view of an aortic perivalvular abscess (arrow). (*C*) Haematoxylin–eosin (H&E)-stained histological section of the mobile vegetation attached to the aortic mechanical prosthesis showing fibrin-rich thrombi and infiltration of neutrophils and macrophages without any evidence of bacteria or tumour tissue. (*D*) Magnified H&E-stained section of mobile vegetation (square in *C*) showing microorganisms that were absent in the thrombotic vegetation.

A 64-year-old man presented to our outpatient clinic with complaints of general fatigue and high fever (>38°C) at night that persisted for 3 days. He had previously undergone 25-mm mechanical aortic valve implantation (St. Jude Medical Regent; St. Jude Medical, Inc., St. Paul, MN) 12 years prior because of severe aortic regurgitation. Upon examination, no focus of infection could be identified, and he was admitted to our heart centre to exclude the possibility of PVE. After three sets of blood culture were obtained, intravenous ceftriaxone (2 g/day) was empirically initiated. Initial transthoracic echocardiography (TTE) revealed no dysfunction of the mechanical aortic prosthesis, and whole-body CT revealed no embolic events. The patient recovered to a low-grade fever (37–38°C) 48 h after the initial antibiotics therapy and showed improved C-reactive protein level (from 13 to 6.3 mg/dL) in the first 5 days. Subsequently, transoesophageal echocardiography (TOE) performed on the 5th day of hospitalization revealed a 6–11 mm mobile echogenic mass attached to the prosthetic valve and a thickened area within the posterior annular region with a low echogenic appearance at the level of the non-coronary sinus of Valsalva (*Figure 1*). At this point, the patient met two minor diagnostic criteria (predisposing heart condition of prior prosthetic valve implantation and high fever) in the modified Duke diagnostic criteria for infective endocarditis because the TOE findings were considered inconclusive. Subsequently, combined ^18^F-FDG PET/CT and CT coronary angiography revealed intense hyper-metabolism above the aortic valve prosthesis with greater intensity at the posterior end and absence of obstructive coronary artery, thereby confirming the diagnosis of aortic PVE complicated with perivalvular abscess (*Figure 2*). Following a comprehensive discussion with the endocarditis team, including an infectious disease specialist, a microbiologist (pathologist) and cardiovascular surgeons, we decided to switch from intravenous ceftriaxone to appropriate antibiotic therapy [intravenous ampicillin (8 g/day) and gentamicin (180 mg/day)] for blood culture-negative patients with PVE based on the current ESC guideline^5^ and withheld specific serological tests or polymerase chain reaction (PCR) assays for unusual but potential microorganisms as long as the patient’s clinical course was not out of track and the antibiotics therapy appeared clinically effective. Following 1 week of intensive antibiotic treatment, we finally decided to perform surgery mainly because the PVE was complicated with perivalvular abscess, as evidenced by ^18^F-FDG PET/CT with all negative blood culture tests. On the 14th day of hospitalization, prosthetic valve was surgically replaced with a 25-mm bioprosthesis (25A Trifecta™; St. Jude Medical, Inc., St. Paul, MN, USA) with extensive debridement of the infected and necrotic tissues surrounding the aortic annulus (*Figure 3A* and *B*). Surgically excised tissue was subjected to microbiological and pathological analysis; however, no evidence of bacterial organisms was found (*Figure 3C* and *D*) and blood culture tests were negative. Post-operative TTE revealed a normally functioning aortic valve, and he was discharged on the 51st day. Based on the international guideline,^5^ antibiotic therapy was continued for 6 weeks until hospital discharge, although gentamicin was discontinued in the first 3 weeks because of nephrotoxicity. Furthermore, intravenous antibiotic treatment was followed by 6-month outpatient oral antibiotic therapy with amoxicillin (750 mg/day) without any recurrences.

## Discussion

The modified Duke criteria have been recommended for PVE diagnosis since its proposal by Durack *et al*.^6^ Notwithstanding the presence of well-established clinical criteria, PVE diagnosis poses some challenges. There is a common concern regarding the interpretation of echocardiographic findings, despite TTE or TOE being the primary diagnostic approach.^7^ As observed in the patient in this study, echocardiographic findings can often be inconclusive, which can be primarily attributed to the similar appearance of both the thrombus and pannus on the prosthetic valves, which cannot be distinguished from vegetation.^7^ This inconclusive diagnosis can lead to difficulty or delay in surgical decisions, particularly in patients with low-to-intermediate likelihood of PVE, such as those with negative blood culture tests. ^18^F-fluorodeoxyglucose positron emission tomography/computed tomography is an established diagnostic modality in patients with suspected PVE that is used when conventional diagnostic tools fail to provide a definitive diagnosis.^5^ In the present patient, perivalvular involvement was a key observation because intense ^18^FDG uptake confined to the aortic annulus above the prosthesis as revealed by ^18^F-FDG PET/CT images was compatible with the thickened area within the aortic annular region observed in TOE examinations, implying perivalvular abscess formation. Additionally, detecting perivalvular involvement by utilizing ^18^F-FDG PET/CT allowed us to improve the diagnostic accuracy of PVE.

As a preoperative risk assessment, CT angiography is more advantageous and safer than invasive coronary angiography in PVE cases involving the aortic valve because invasive coronary angiography potentially involves risks of vegetation embolization or haemodynamic decompensation. Also, CT angiography can provide further information on the valve size, anatomy, and calcification of aortic valve, which are essential for surgical planning.^8^ Furthermore, compared with the TOE findings, the combination of PET and CT angiography enhances the accuracy of identification of the infected location, in addition to the delineation of coronary anatomy and detection of fistulae and abscess, distal embolic complications and infected aneurysms at other sites.^4^

Another important case discussion was on how to manage the blood culture-negative patient with PVE with appropriate antibiotic therapy and/or surgical intervention if needed. The patient was initially treated with ceftriaxone in empirical therapy for unexplained fever following three sets of blood culture tests. Although the patient’s condition stabilised in the first week, with improvement in fever and general appearance, we switched ceftriaxone to intravenous ampicillin and gentamicin because the findings of ^18^F-FDG PET/CT imaging were highly suggestive of PVE. These antibiotic regimens for initial empirical treatment of late PVE were determined based on the current ESC guideline^5^ and consultation with the endocarditis team. In a recent review,^9^ Viridans group *streptococci*, coagulase-negative *staphylococci* (CNS), *Enterococcus* spp. and *Streptococcus bovis* were reported to be the most common pathogens in patients with PVE. We carefully selected a combination of ampicillin and gentamicin to mainly treat *staphylococci*, *streptococci,* and *enterococci*. We assumed that the patient’s clinical course was relatively subacute and less destructive with no major complications such as heart failure and systematic embolism and ceftriaxone seemed to be clinically effective. Considering this, PVE triggered by *Staphylococcus aureus* or CNS was slightly less likely to occur in the patient. Blood culture-negative infective endocarditis (BCNIE) can be caused by fungi or fastidious slow-growing bacteria, notably obligatory intracellular bacteria.^1^ Among them, nutritionally variant *Streptococcus* spp. and HACEK (Haemophilus, Aggregatibacter, Cardiobacterium, Eikenella, and Kingella) could be the potential causative microbes in the present case; therefore, we selected a combination of ampicillin and gentamicin for treatment based on the local epidemiology. Indeed, we were ready to perform systemic serological testing for less common microorganisms in BCNIE patients, such as *Coxiella burnetii*, *Bartonella* spp., *Aspergillus* spp., *Mycoplasma pneumonia*, *Brucella* spp., and *Legionella pneumophila*, followed by specific PCR assays. However, we kept the samples and did not perform these tests because the patient’s condition had gradually recovering after the initiation of intravenous ampicillin and gentamicin during the whole hospital stay, including the perioperative period. Additionally, both the blood and tissue PCR tests, which are not covered by Japan’s national health insurance, generally take a considerable amount of time and cost. A 6-month outpatient oral antibiotic therapy was completed because we emphasized on the remarkable findings: PVE was complicated with perivalvular abscess formation and unidentified microorganisms.

In summary, we report a case regarding a patient with blood culture-negative PVE complicated with perivalvular abscess, with a low-to-intermediate likelihood of PVE based on the modified Duke criteria, in whom ^18^F-FDG PET/CT successfully provided a definitive diagnosis of perivalvular abscess and infective endocarditis, leading to subsequent surgical intervention within an appropriate timeframe.

## Lead author biography

**Figure:**
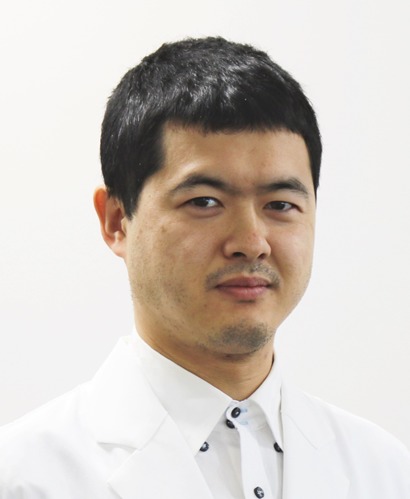


Dr Shiro Miura is working as a chief interventional cardiologist at one of the most outstanding cardiovascular centers in Hokkaido in Japan named Hokkaido Ohno Memorial Hospital with remarkable achievements specializing in interventions on ischemic heart diseases and structural heart disease in the management of inpatients and outpatients with heart failure following the postgraduate course named ‘MSc Statistics with Applications in Medicine’ in University of Southampton in the UK where I was rewarded the Dean’s List Awards for exceptional performance for both the regular term exams and final dissertation.

## Supplementary material

Supplementary material is available at *European Heart Journal - Case Reports* online.

**Slide sets:** A fully edited slide set detailing this case and suitable for local presentation is available online as Supplementary data.

**Consent:** The author/s confirm that written consent for submission and publication of this case report including image(s) and associated text has been obtained from the patient in line with COPE guidance. 

**Conflict of interest:** none declared.

## Supplementary Material

ytz171_Supplementary_Slide_Set
