# Supplementary material for: Usefulness of 18F-fluorodeoxyglucose positron emission tomography/computed tomography angiography in a patient with blood culture-negative prosthetic valve endocarditis complicated with perivalvular abscess: a case report
Source: Eur Heart J Case Rep. 2019 Oct 11;3(4):1–5. doi: 10.1093/ehjcr/ytz171 (PMC6939801; doi:10.1093/ehjcr/ytz171)
Supplement: ytz171_Supplementary_Slide_Set [file ytz171_supplementary_slide_set.pptx]

## Slide 1
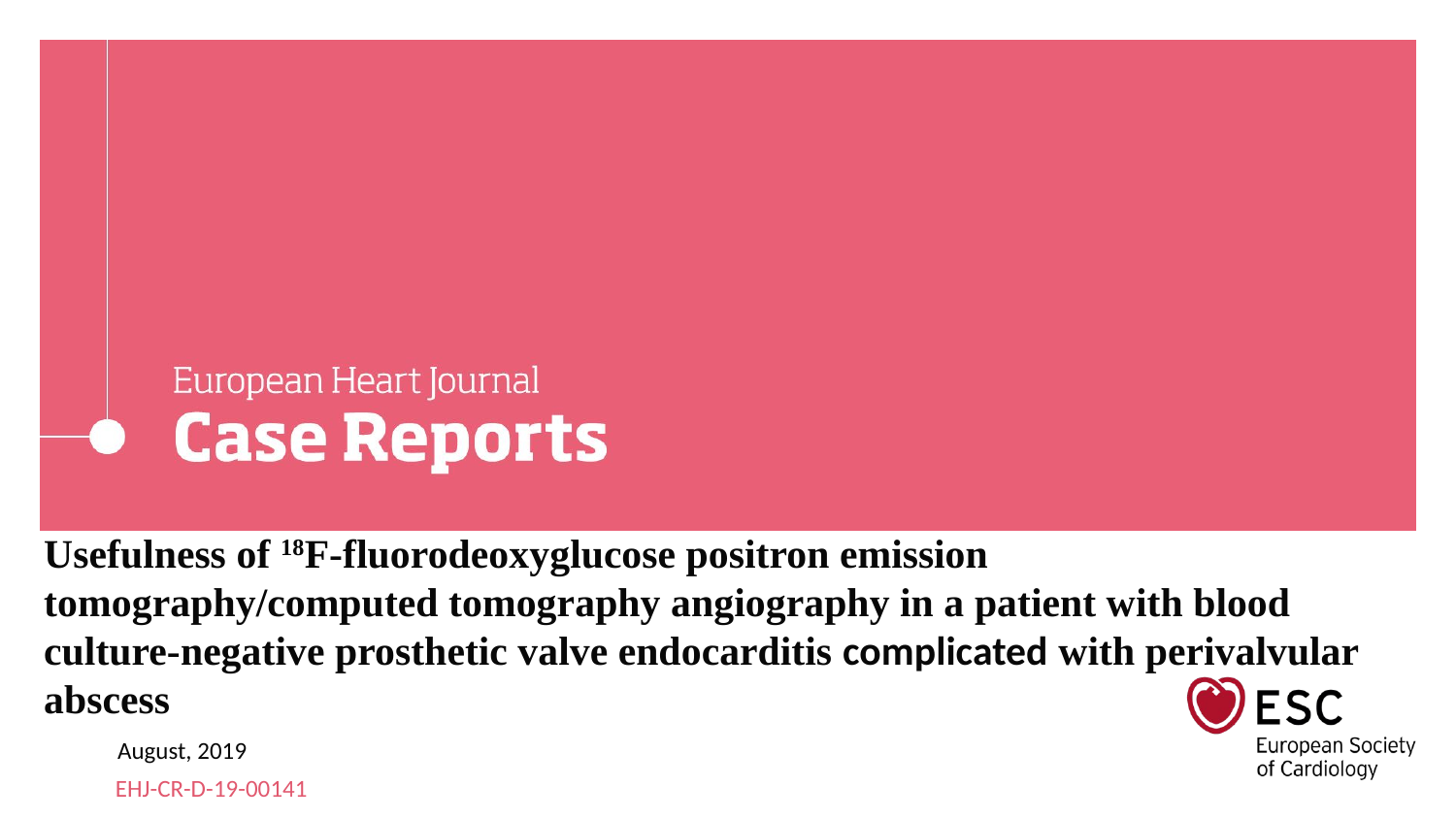

# Usefulness of 18F-fluorodeoxyglucose positron emission tomography/computed tomography angiography in a patient with blood culture-negative prosthetic valve endocarditis complicated with perivalvular abscess
August, 2019
EHJ-CR-D-19-00141

## Slide 2
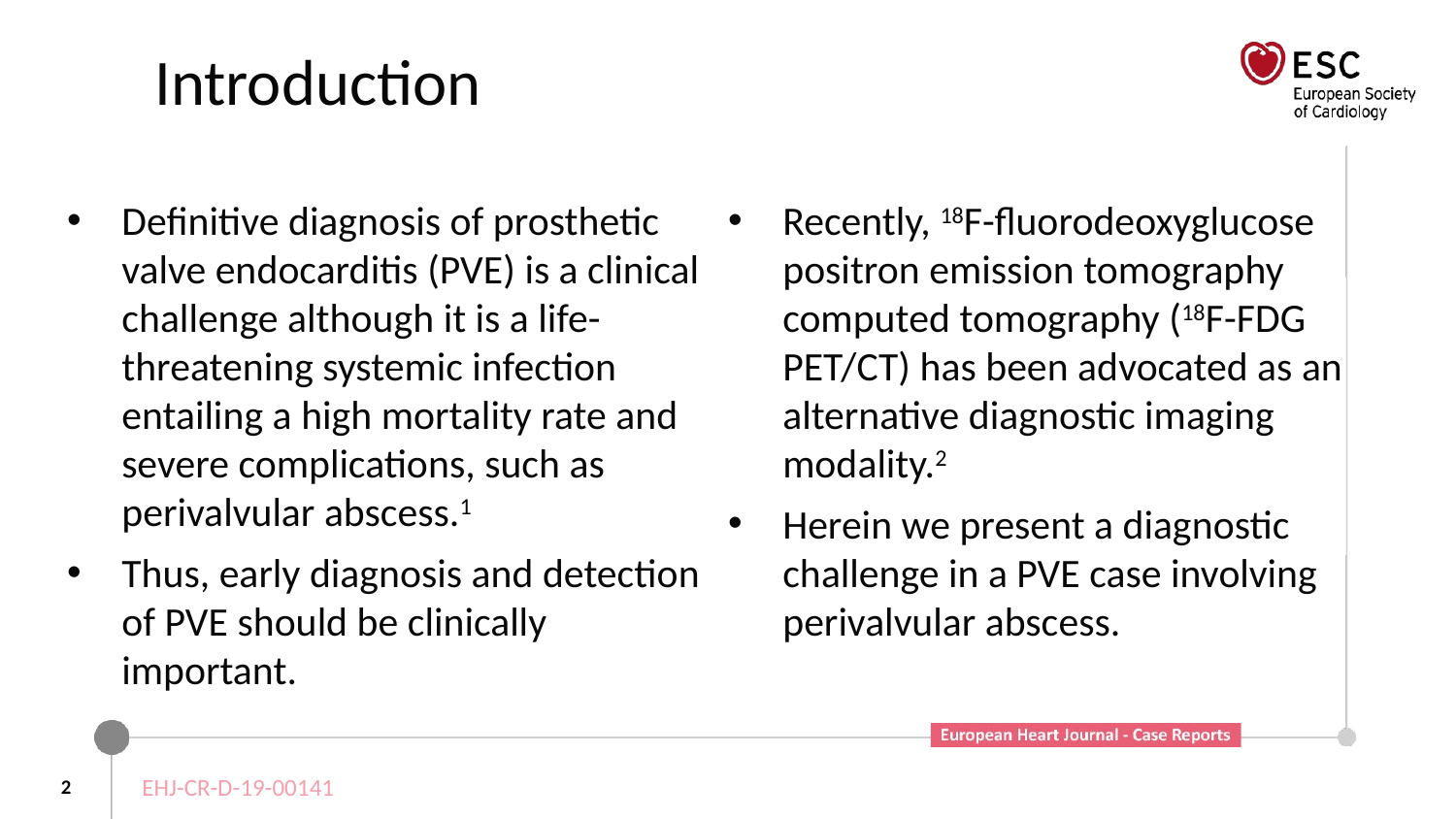

# Introduction
Definitive diagnosis of prosthetic valve endocarditis (PVE) is a clinical challenge although it is a life-threatening systemic infection entailing a high mortality rate and severe complications, such as perivalvular abscess.1
Thus, early diagnosis and detection of PVE should be clinically important.
Recently, 18F-fluorodeoxyglucose positron emission tomography computed tomography (18F-FDG PET/CT) has been advocated as an alternative diagnostic imaging modality.2
Herein we present a diagnostic challenge in a PVE case involving perivalvular abscess.
2
EHJ-CR-D-19-00141

## Slide 3
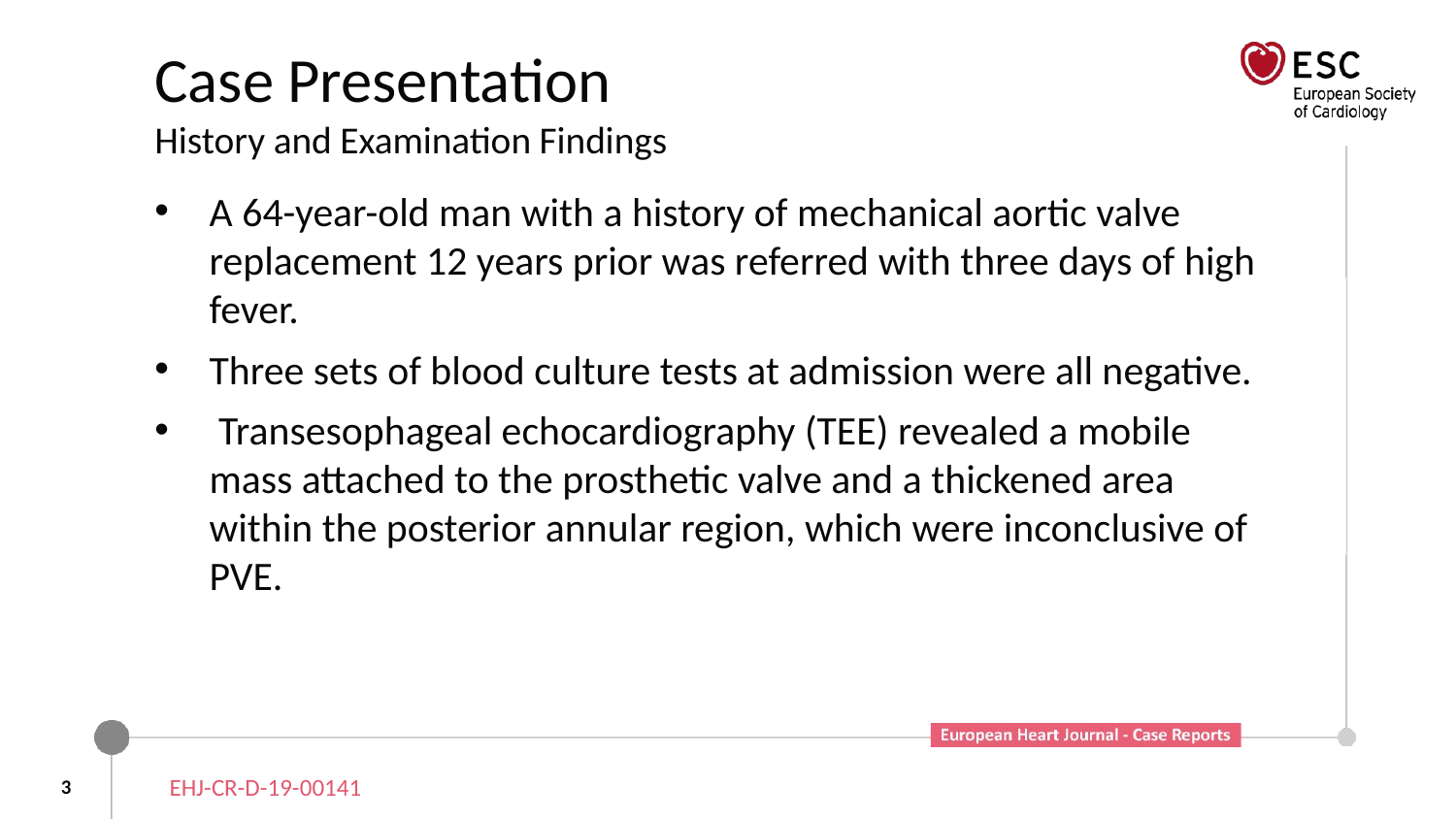

# Case Presentation History and Examination Findings
A 64-year-old man with a history of mechanical aortic valve replacement 12 years prior was referred with three days of high fever.
Three sets of blood culture tests at admission were all negative.
 Transesophageal echocardiography (TEE) revealed a mobile mass attached to the prosthetic valve and a thickened area within the posterior annular region, which were inconclusive of PVE.
3
EHJ-CR-D-19-00141

## Slide 4
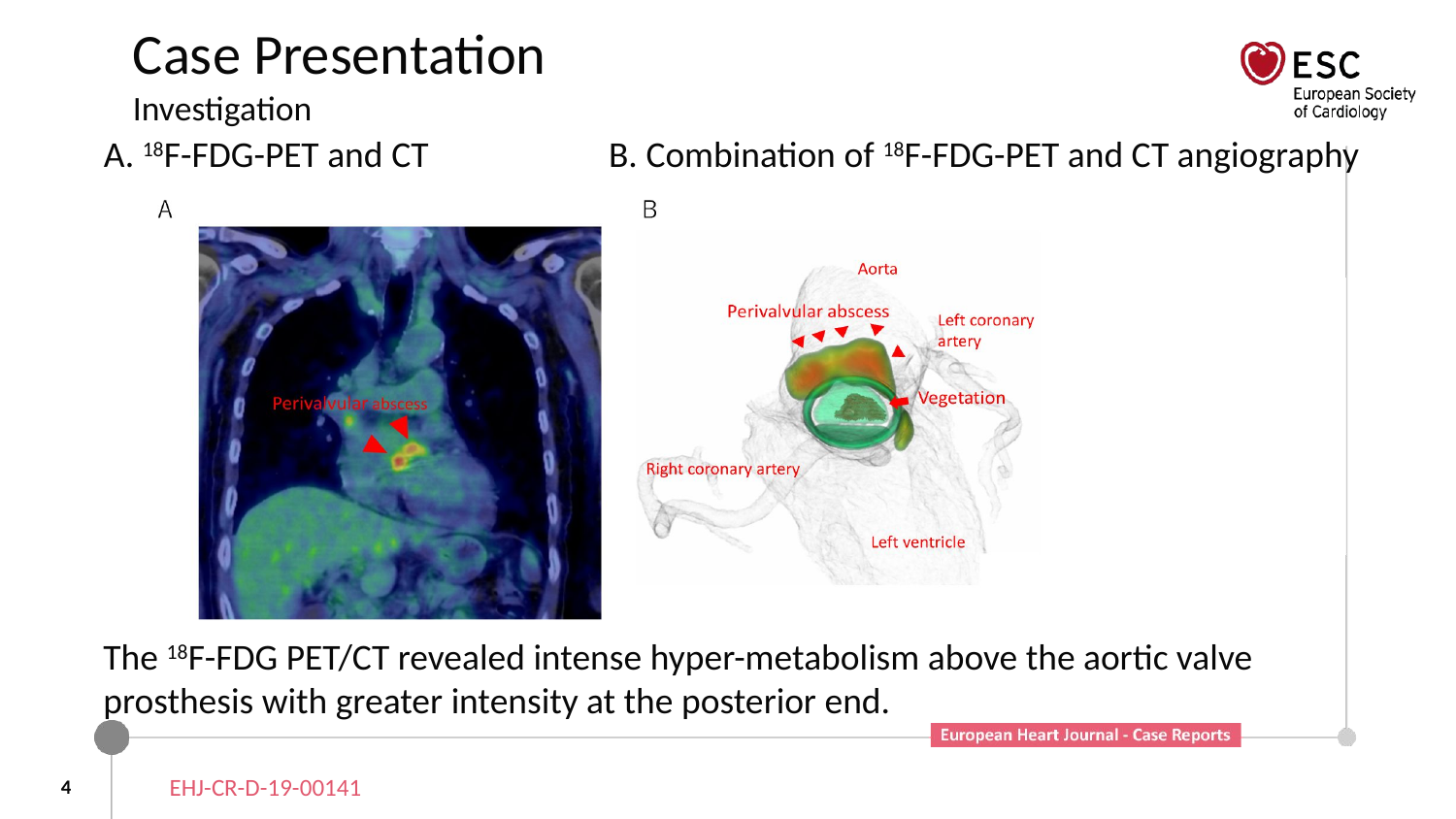

Case Presentation
Investigation
A. 18F-FDG-PET and CT
B. Combination of 18F-FDG-PET and CT angiography
The 18F-FDG PET/CT revealed intense hyper-metabolism above the aortic valve prosthesis with greater intensity at the posterior end.
4
EHJ-CR-D-19-00141

## Slide 5
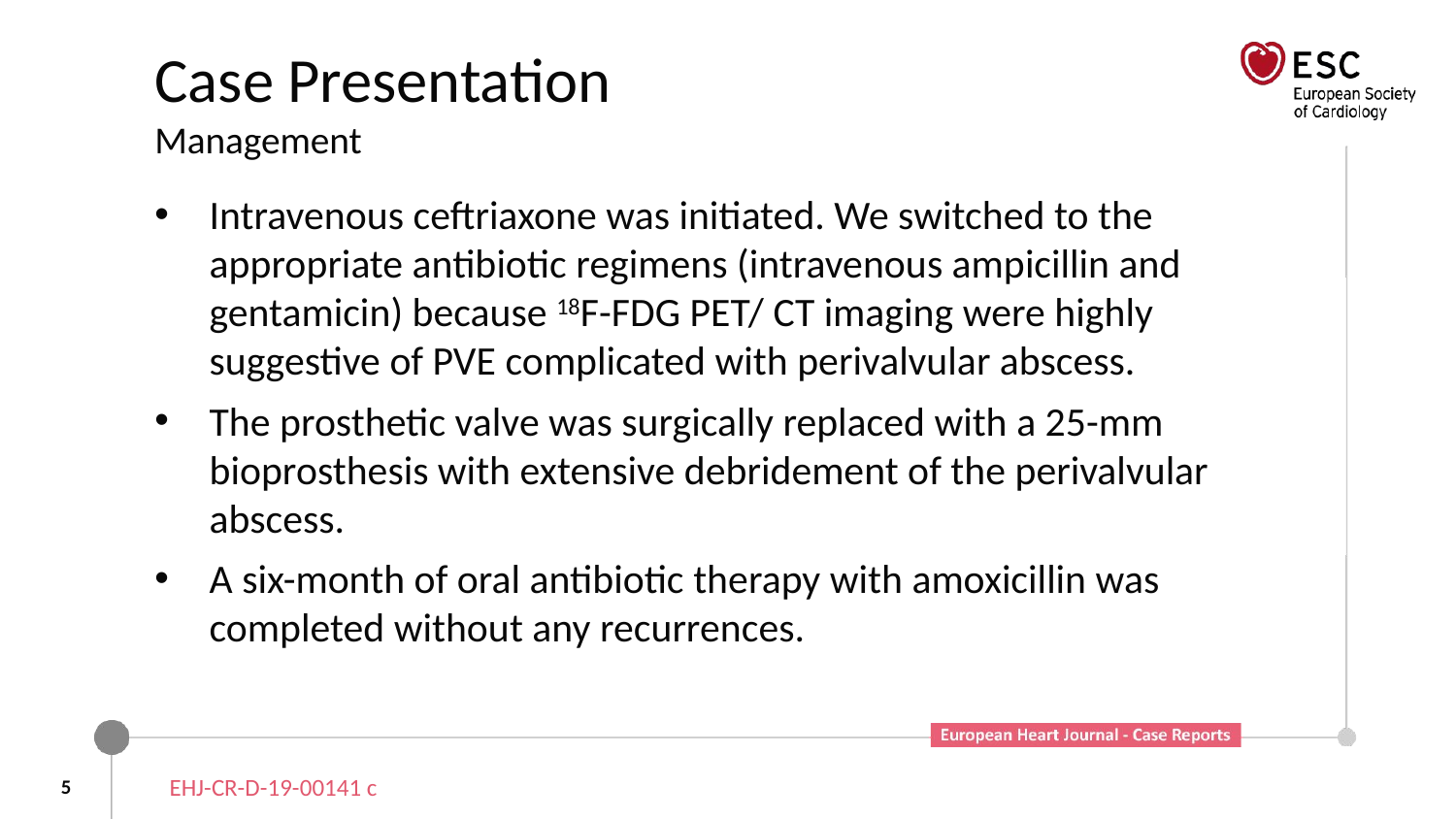

# Case Presentation Management
Intravenous ceftriaxone was initiated. We switched to the appropriate antibiotic regimens (intravenous ampicillin and gentamicin) because 18F-FDG PET/ CT imaging were highly suggestive of PVE complicated with perivalvular abscess.
The prosthetic valve was surgically replaced with a 25-mm bioprosthesis with extensive debridement of the perivalvular abscess.
A six-month of oral antibiotic therapy with amoxicillin was completed without any recurrences.
5
EHJ-CR-D-19-00141 c

## Slide 6
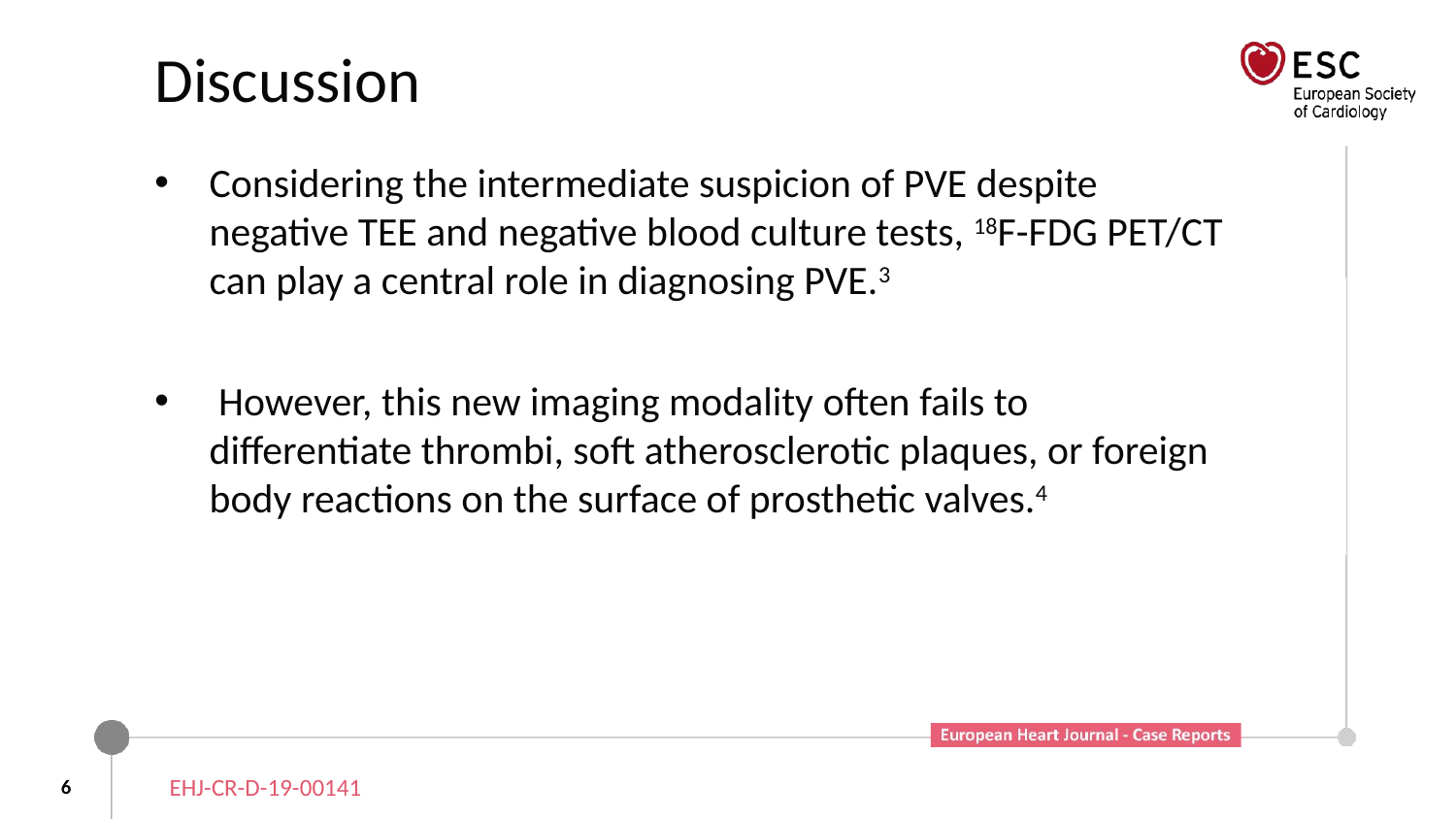

# Discussion
Considering the intermediate suspicion of PVE despite negative TEE and negative blood culture tests, 18F-FDG PET/CT can play a central role in diagnosing PVE.3
 However, this new imaging modality often fails to differentiate thrombi, soft atherosclerotic plaques, or foreign body reactions on the surface of prosthetic valves.4
6
EHJ-CR-D-19-00141

## Slide 7
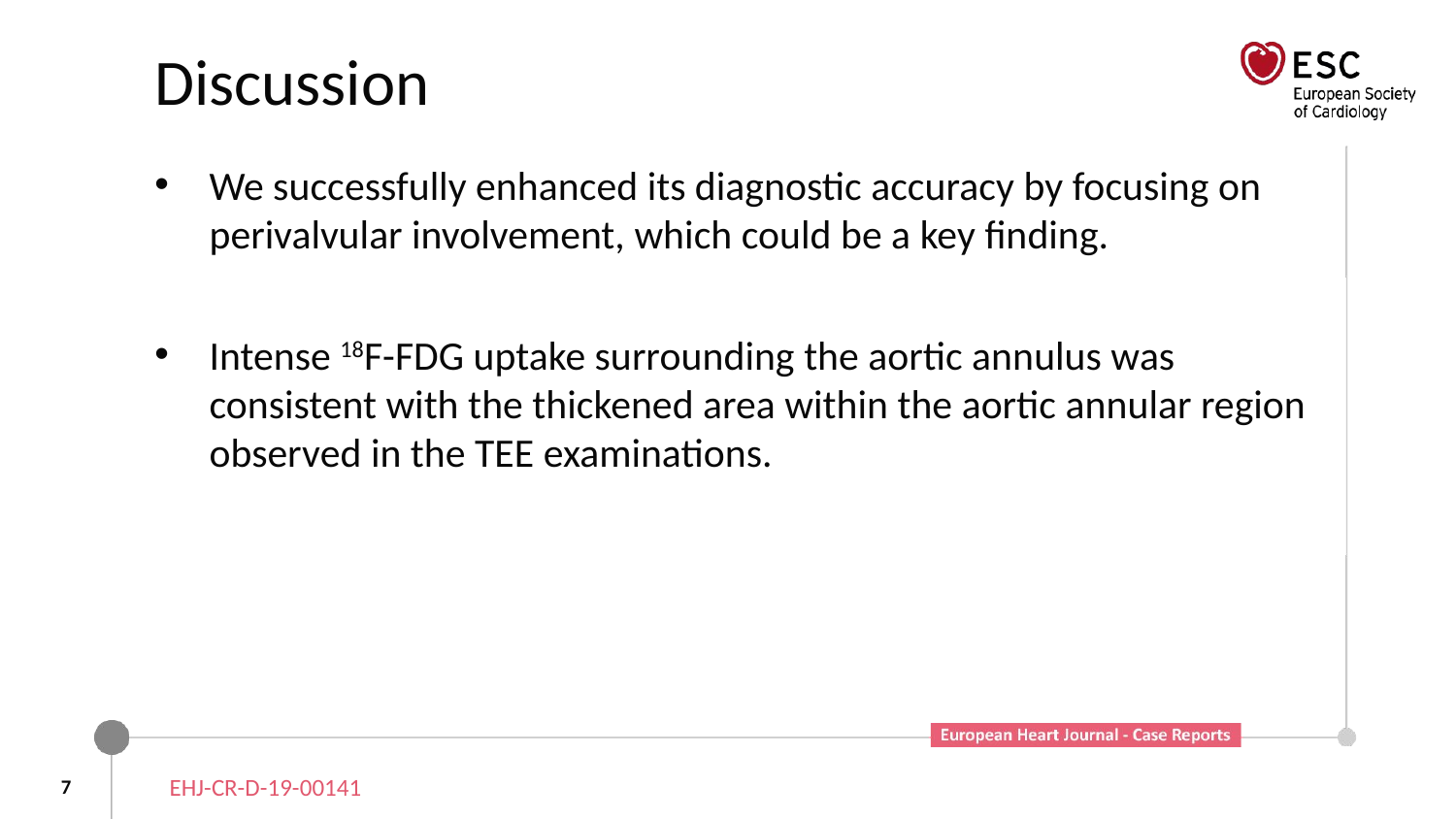

# Discussion
We successfully enhanced its diagnostic accuracy by focusing on perivalvular involvement, which could be a key finding.
Intense 18F-FDG uptake surrounding the aortic annulus was consistent with the thickened area within the aortic annular region observed in the TEE examinations.
7
EHJ-CR-D-19-00141

## Slide 8
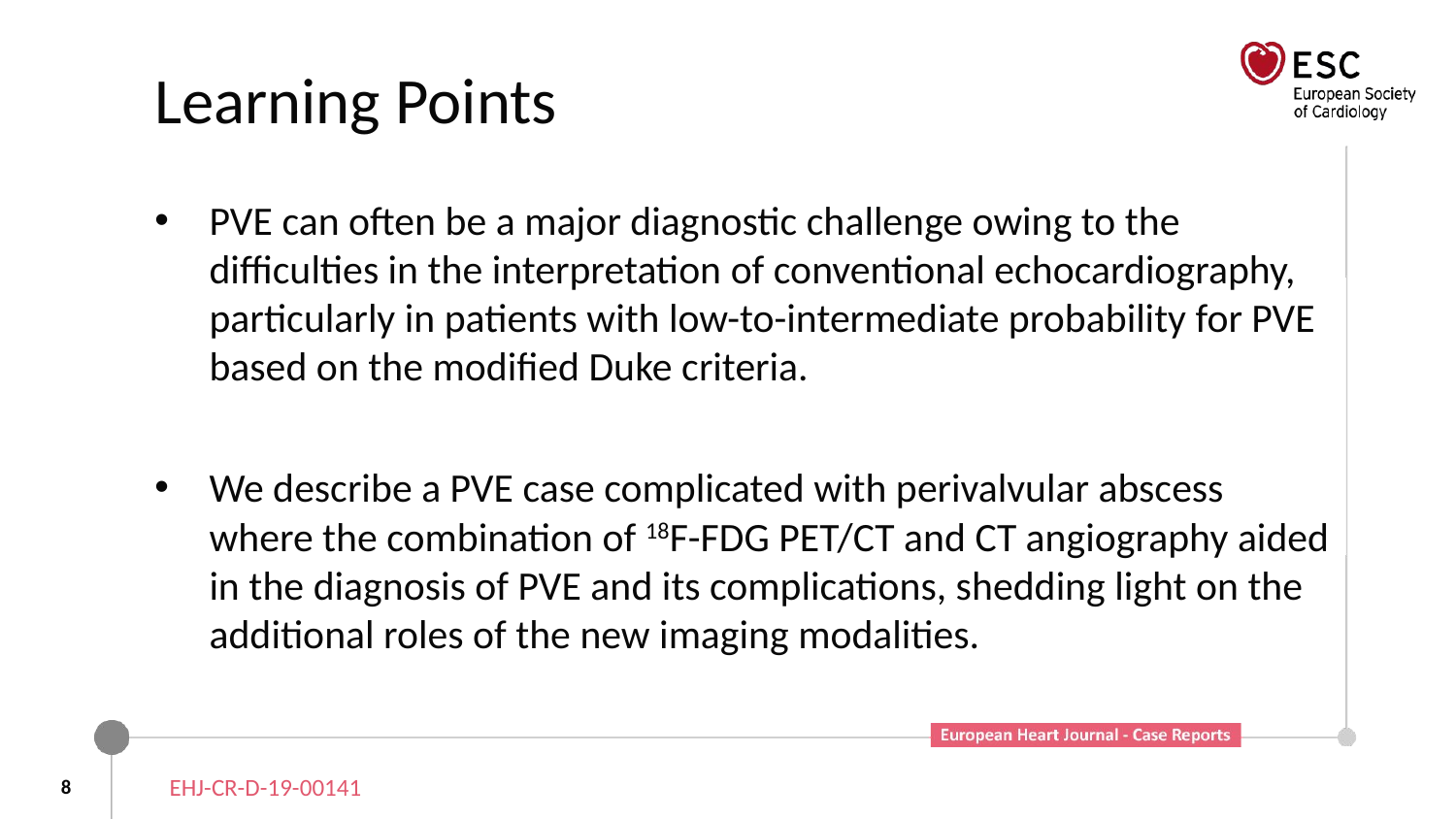

# Learning Points
PVE can often be a major diagnostic challenge owing to the difficulties in the interpretation of conventional echocardiography, particularly in patients with low-to-intermediate probability for PVE based on the modified Duke criteria.
We describe a PVE case complicated with perivalvular abscess where the combination of 18F-FDG PET/CT and CT angiography aided in the diagnosis of PVE and its complications, shedding light on the additional roles of the new imaging modalities.
8
EHJ-CR-D-19-00141

## Slide 9
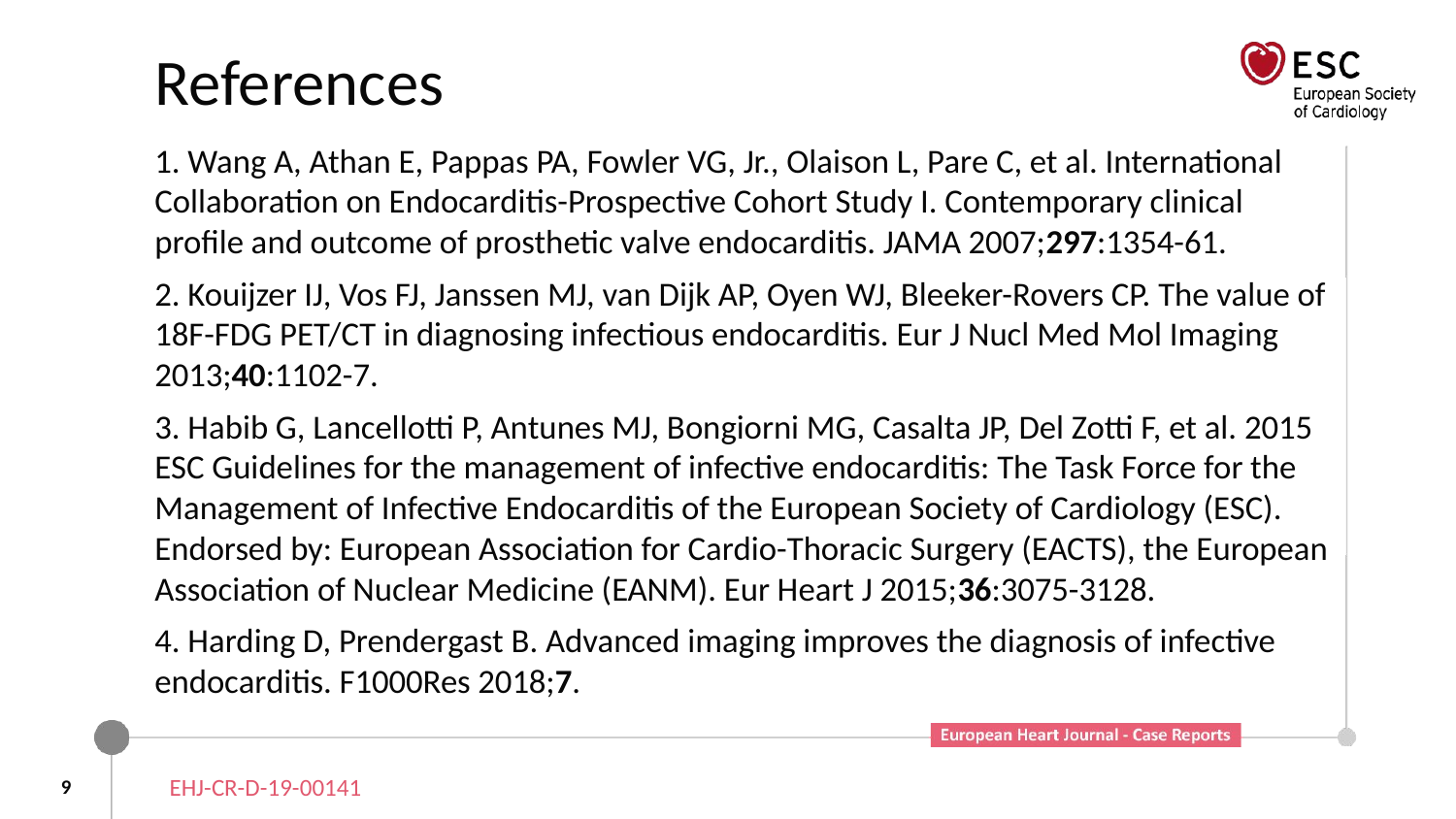

# References
1. Wang A, Athan E, Pappas PA, Fowler VG, Jr., Olaison L, Pare C, et al. International Collaboration on Endocarditis-Prospective Cohort Study I. Contemporary clinical profile and outcome of prosthetic valve endocarditis. JAMA 2007;297:1354-61.
2. Kouijzer IJ, Vos FJ, Janssen MJ, van Dijk AP, Oyen WJ, Bleeker-Rovers CP. The value of 18F-FDG PET/CT in diagnosing infectious endocarditis. Eur J Nucl Med Mol Imaging 2013;40:1102-7.
3. Habib G, Lancellotti P, Antunes MJ, Bongiorni MG, Casalta JP, Del Zotti F, et al. 2015 ESC Guidelines for the management of infective endocarditis: The Task Force for the Management of Infective Endocarditis of the European Society of Cardiology (ESC). Endorsed by: European Association for Cardio-Thoracic Surgery (EACTS), the European Association of Nuclear Medicine (EANM). Eur Heart J 2015;36:3075-3128.
4. Harding D, Prendergast B. Advanced imaging improves the diagnosis of infective endocarditis. F1000Res 2018;7.
9
EHJ-CR-D-19-00141
